# A genome-wide association study on liver enzymes in Korean population

**DOI:** 10.1371/journal.pone.0229374

**Published:** 2020-02-21

**Authors:** Ji Yeon Seo, Jong-Eun Lee, Goh Eun Chung, Eunsoon Shin, Min-Sun Kwak, Jong In Yang, Jeong Yoon Yim

**Affiliations:** 1 Department of Internal Medicine, Gangnam Healthcare Center, Seoul National University Hospital, Seoul, Korea; 2 DNALink, Inc., Seoul, Korea; National Institute of Environmental Health Sciences, UNITED STATES

## Abstract

**Background:**

Although genetic features vary across ethnicities, few genome-wide association studies (GWAS) have reported the genetic determinants of liver enzyme expression. This study was aimed to evaluate the associations of genome-wide single nuclear polymorphisms (SNPs) with the liver enzymes in a Korean population.

**Methods:**

We performed a GWAS to identify genetic loci influencing liver function, as measured by concentrations of alkaline phosphatase (ALP), alanine transaminase (ALT), gamma-glutamyl transferase (GGT) and total bilirubin (BIL) in in Korean study participants.

**Results:**

A total of 6,488 subjects (4,457 in the discovery and 2,031 in the validation set) were included. The mean subject age was 50.0±10.6 years (male, 53.7%). Among a total of 546,738 SNPs tested, rs651007 and rs579459 located in the *ABO* gene showed strong associations with ALP (*P* = 1.63×10^−8^ and 5.61×10^−8^, respectively [discovery set]; *P* = 4.08×10^−15^ and 9.92×10^−16^, respectively [validation set]). Additionally, rs5751901 and rs2006092, which are located in the *GGT1* gene, showed strong associations with GGT (*P* = 6.44×10^−15^ and 1.26×10^−15^, respectively [discovery set]; *P* = 4.13×10^−10^ and 5.15×10^−11^, respectively [validation set]). Among the 13 SNPs that showed genome-wide significance with total bilirubin levels, rs10929302 and rs6742078 showed the most significant association (*P* = 3.08×10^−64^ and 2.05×10^−62^, respectively [discovery set]; *P* = 1.33×10^−116^ and 2.24×10^−118^, respectively [validation set]). No genome-wide significant associations was found for ALT.

**Conclusions:**

We demonstrated that *ABO*, *GGT1* and *UGT1A* family were associated with ALP, GGT and BIL, respectively in Korean population. These findings differ from reported results in GWAS in European populations in terms of associated genes and locations, suggesting different genetic mechanisms of liver enzyme regulation according to ethnicity.

## Introduction

Liver function tests (LFTs) are commonly used in clinic approaching hepatobiliary disease. These tests offers clinical information on the presence, severity and mortality of liver disease, and differential diagnosis of other diseases. They are also necessary for monitoring and determining of response to treatment [1, 2]. However, careful interpretation is required because LFT could appear normal in advanced liver disease and abnormal LFTs could reflect other disease such as cardiovascular disease, diabetes, or metabolic syndrome [3, 4].

Plasma levels of liver enzymes vary from person to person, which is affected by both environmental and genetic factors. The hereditability of LFT levels are estimated from 22–60% [5, 6], suggesting a genetic role in interpretation of the results. Single-nucleotide polymorphism (SNP) is useful to analyze genetic elements of clinical features. Several genome-wide association studies (GWAS) were reported to find out genetic mechanisms of LFT variations. Most frequently reported gene associated with gamma-glutamyl transferase (GGT) is GGT1, which is involved in glutathione metabolism. *HNF1A* gene, related to lipid metabolism and inflammation, is also reported [7–9]. *PNPLA3* gene, involved in energy mobilization and storage of adipocyte, has been reported several times to be related to both aspartate aminotransferase (AST) and alanine-aminotransferase (ALT) [7–11]. *CHUK* gene, related to glucose and lipid metabolism, is also known to be significant gene influencing ALT level [7, 8]. In addition, *ALPL*, *GPLD1*, and *JMJD1C-REEP3* gene and *ABO* locus are appeared to have significant relationship with alkaline phosphatase (ALP) level [7]. For bilirubin, associations with various SNPs in *UGT1A1* gene is reported. *UGT1A1* is involved in conjugation of bilirubin and polymorphism of *UGT1A1* is associated with Gilbert syndrome [12–15]. Other various genes associated with liver function tests has been reported, however the result varies according to the study population.

Although genetic features are known to differ between ethnicities, few studies on Asians have been published. Korea is endemic area of hepatitis B virus and incidence of nonalcoholic fatty liver disease (NAFLD) is increasing. Therefore, understanding genetic features of LFT in Korean population is important. This study was aimed to evaluate the associations of genome-wide SNPs with the liver enzymes in a Korean population.

## Patients and methods

### Study population

For our study, we analyzed the database from a previously described GENIE cohort [16]. Briefly, 8,000 people donated blood samples during a routine health check-up program at Seoul National University Hospital Healthcare System Gangnam Center between January 2014 and December 2014, and their blood samples were stored at a biorepository with their informed consent.

Subjects with significant alcohol intake (>20 g/day for males and >10 g/day for females) were excluded (n = 842). We also excluded 248 individuals who were positive for hepatitis B virus and 48 subjects who were positive for hepatitis C virus. Additionally, subjects with missing information were excluded from the study. This study was approved by the Institutional Review Board of the Seoul National University Hospital with a waiver of informed consent (No 1908-126-105).

### Clinical and laboratory assessments

The methods employed in this study have been described in detail elsewhere [17]. Briefly, each subject completed a past medical history questionnaire and underwent anthropometric assessment. The body weight and height of each subject were measured using a digital scale. Body mass index (BMI) was calculated as the ratio of weight (kg) to area (m^2^, estimated from height). Waist circumference was measured at the midpoint between the lower costal margin and the anterior superior iliac crest by a well-trained person. Systolic blood pressure and diastolic blood pressure were each measured twice, and their mean values were reported. After an overnight 12h period, blood samples were collected from the antecubital vein of each individual. The laboratory evaluations included serum ALT, AST, ALP, GGT, total bilirubin (BIL) total cholesterol, triglyceride, fasting glucose, hepatitis B surface antigen and antibody to hepatitis C virus. Hypertension was defined as having systolic blood pressure ≥140 mmHg, having diastolic blood pressure ≥90 mmHg or using of anti-hypertensive medication. Diabetes mellitus was defined as either a fasting serum glucose level of ≥126 mg/dL or the use of anti-diabetic medication.

### Genome-wide genotyping and quality control

Genomic DNA was isolated from venous blood samples and 200 ng of DNA from each patient was genotyped using Affymetrix Axiom^®^ Customized Biobank Genotyping Arrays (Affymetrix, Santa Clara, CA, USA). The PLINK program (version 1.90, Free Software Foundation Inc., Boston, MA, USA) was used for quality control procedures. Samples meeting any of the following criteria were removed: (i) gender inconsistency, (ii) call rate ≤ 97% and (iii) related and cryptically related individuals (identical by descent >0.185). SNPs were filtered if (1) the call rate was <95%, (2) the minor allele frequency (MAF) was ≤ 0.01, or (3) there was a significant deviation from the Hardy-Weinberg equilibrium permutation test (*P*<1x10^-4^).

### Statistical analysis

Linear-regression analyses assuming and additive genetic model was used on serum ALP, ALT, GGT and total bilirubin using PLINK software package to test the association of liver enzymes with SNPs in the genome. Age, and gender were used as covariates. The R statistical software package (version 3.1.1, R development Core Team; R Foundation for Statistical Computing, Vienna, Austria) was used for the statistical analysis and to draw the Manhattan plot of–log10. The quantile-quantile plots were generated using R statistics package to assess the overall significance of the genome-wide associations. The LocusZoom (http://csg.sph.umich.edu/locuszoom) was used to graphically display the genome-wide association scan results. In the regional plot, hg19/1000 Genomes Nov 2014 ASN was used as the reference panel for linkage disequilibrium [18].

The results were verified using discovery and validation sets. We divided the enrolled population into 2 groups based on the time of enrollment. Samples from subjects who enrolled from January, 2014 to October, 2014 were used as the discovery set, and samples from subjects who enrolled in the subsequent months were used as the validation set. SNPs that had a *P-*value of less than 1x10^-7^ (by the Bonferroni correction method) in the discovery set were re-evaluated in the validation set. Comparisons of continuous variables between the two groups were performed using Student’s *t*-test, and categorical variables were compared using a chi-square test or Fisher’s exact test. *P-*values of less than 0.05 in the validation set were considered significant.

## Results

### Study population

A total of 6,488 subjects were included. The mean subject age was 50.0±10.6 years, and 53.7% of the subjects were male. Based on the definition described in the methods, there were 4,457 samples in the discovery set and 2,031 samples in the validation set. The characteristics of the study population are described in Table 1. The subjects in the validation set showed greater proportions of male, higher diastolic pressure, BMI ALP, ALT, GGT and fasting glucose (*P* <0.05).

**Table 1:** Baseline characteristics of the study population.

	Discovery set (N = 4,457)	Validation set (N = 2,031)	*P*-Value
Age (years)	50.1±10.6	50.0±10.5	0.102
Male, *n* (%)	2344(52.6%)	1140(56.1%)	0.008
Diabetes mellitus, *n* (%)	254 (5.7)	135 (6.6)	0.136
Hypertension, *n* (%)	821(18.4)	398(19.6)	0.261
Systolic blood pressure (mmHg)	114.6±13.3	115.3±13.1	0.052
Diastolic blood pressure (mmHg)	74.6±11.3	75.9±10.3	<0.001
Body mass index (kg/m^2^)	22.9±3.0	23.1±3.0	0.002
Waist circumference (cm)	81.1±11.9	81.4±11.0	0.435
alkaline phosphatase (mg/dL)	52.7±16.0	55.3±15.9	<0.001
ALT (IU/L)	22.1±16.0	23.1±16.9	0.015
Total bilirubin (mg/dL)	0.92 ± 0.90	0.94±0.82	0.398
GGT (mg/dL)	29.7± 28.1	32.0±32.0	0.005
Total cholesterol (mg/dL)	192.6±33.7	194.1±34.3	0.096
Triglyceride (mg/dL)	103.3±70.6	105.4±71.7	0.265
Fasting glucose (mg/dL)	97.1±15.3	98.7±18.4	<0.001

Data are shown as the mean ± SD.

ALT; alanine-aminotransferase, AST; aspartate aminotransferase, GGT; gamma-glutamyl transferase

### Genome-wide association of live enzymes

**Fig 1:**
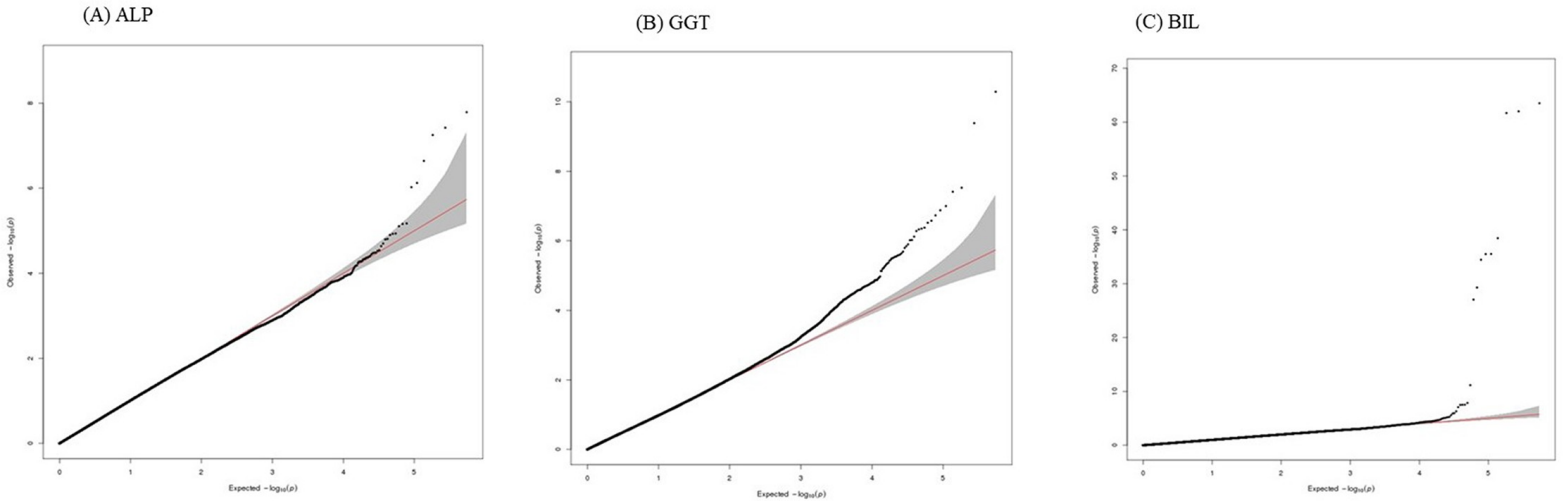
Quantile–quantile plots of association results for GGT (A), ALP (B) and BIL (C). X-axis and Y-axis indicate the negative log-scale of expected p-values of each SNP and the negative log-scale of the observed p-values, respectively. A straight line indicates the expected results under Hardy-Weinberg equilibrium.

A total of 546,738 autosomal SNPs were tested for the association analysis. Quantile–quantile plots (Fig 1) revealed the presence of a substantial number of SNPs associated with ALP, GGT, ALT and BIL levels at a genome-wide significance level (*P* < 10^−7^).

**Fig 2:**
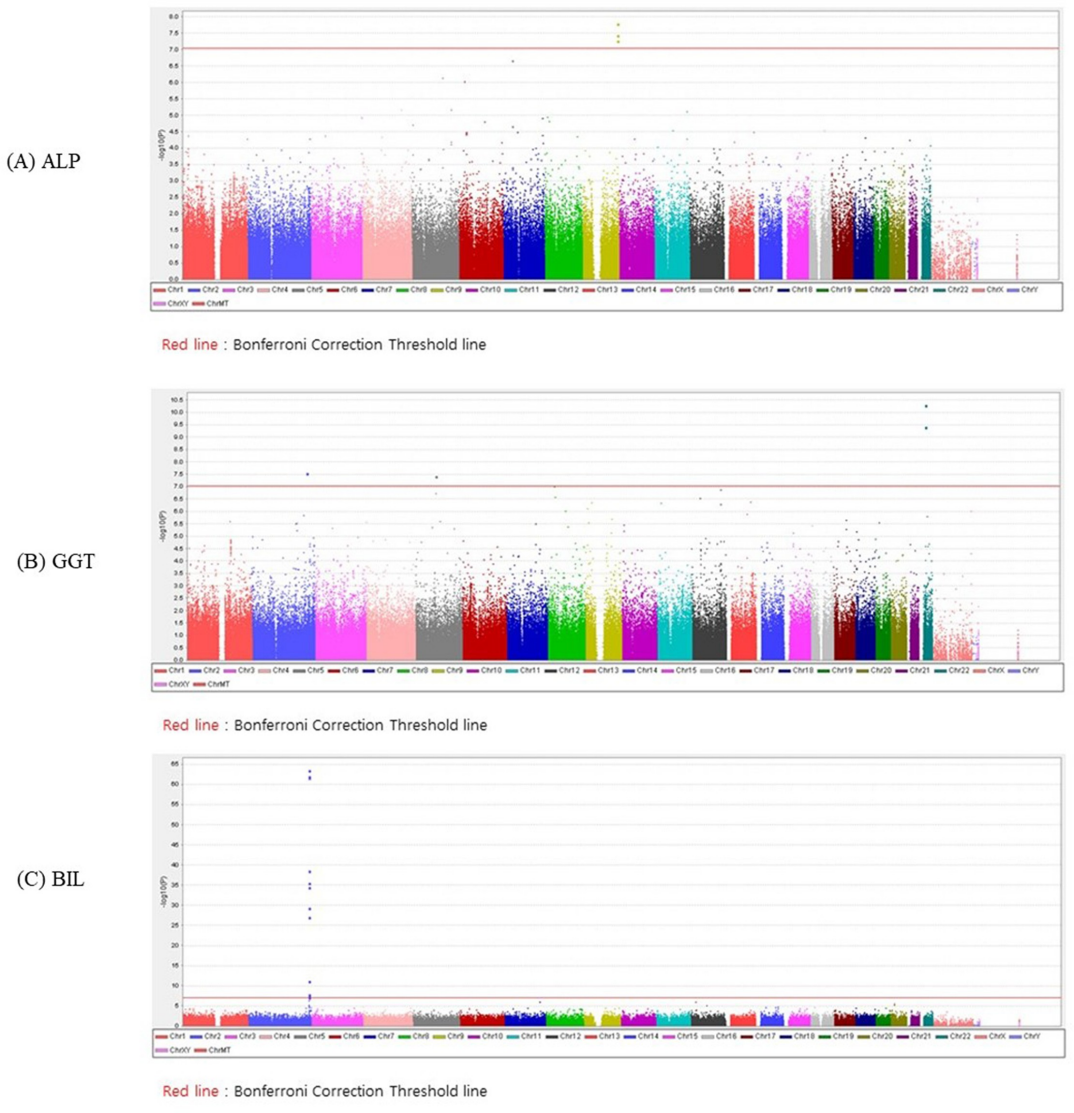
Manhattan plot of genome-wide association signals with liver in the discovery set. (A) ALP (B) GGT and (C) BIL. In the Manhattan plot, the x-axis represents the SNP markers on each chromosome. The y-axis shows the -log10 p-value (logistic regression). The red horizontal line represents the genome-wide significance threshold, and the blue horizontal line represents the genome-wide suggestiveness threshold.

**Fig 3:**
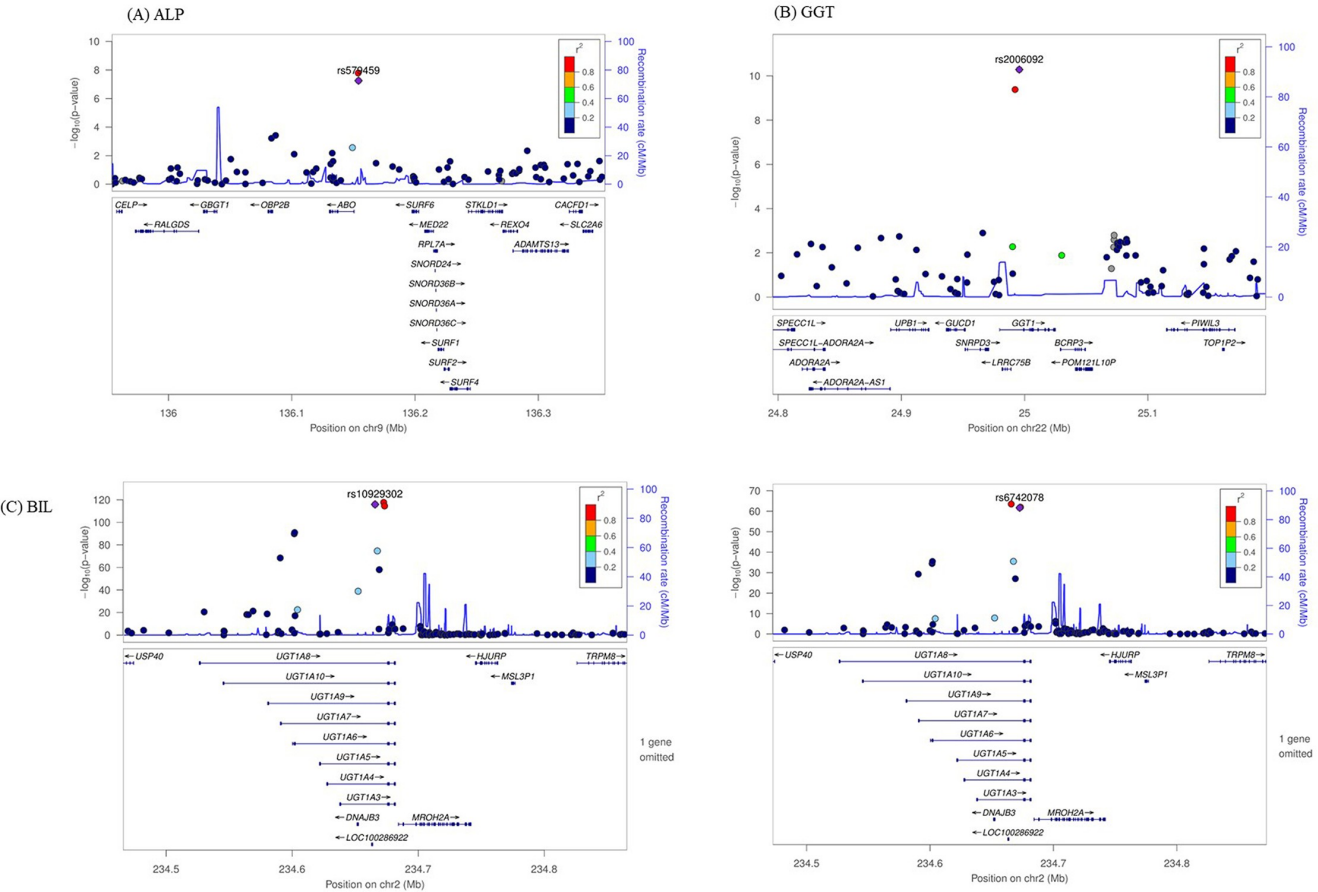
Regional association plots of (A) ALP (B) GGT and (C) BIL. The purple diamonds indicate the associated SNP according to joint analyses. Nearby SNPs are color-coded according to the level of linkage disequilibrium with the top SNP. The left y-axis shows the significance of the association on a -log10 p-value (logistic regression), and the right y-axis shows the recombination rate across the region. Estimated recombination rates from the 1000 Genomes Project Asian base data and hg19 database^16^ are plotted with the blue line to reflect the local linkage disequilibrium structure.

We first evaluated genome-wide associations in the discovery set, with a significance threshold of *P* < 1x10^-7^ as the threshold after adjusting for age and gender. Fig 2 shows Manhattan plots showing SNPs that were associated with liver enzymes (A) ALP (B) GGT and (C) BIL. After joint analysis of discovery and replication set, 3 for ALP, 2 for GGT and 13 for BIL reached genome-wide significance (Table 2). In the discovery set of the GWAS, 11 SNPs were significantly associated with ALP and we performed a validation test, 3 remained significant in the validation set. Among them, 2 SNPs, namely, rs651007 and rs579459 located in the *ABO* gene showed strong associations with ALP (*P*-values, discovery set = 1.63 x10^-8^, and 5.61 x10^-8^, respectively; validation set = 4.08 x10^-15^, and 9.92 x10^-16^, respectively). In the regional plot in chromosome 9 is provided in Fig 3(A), which shows rs579459 as the top SNP and nearby SNPs with the level of high linkage disequilibrium (LD) with rs579459.

**Table 2:** Genetic loci associated with liver enzymes at p < 1 × 10−^7^ in the GWAS of Korean.

					Discovery			Validation		
SNP	Chr	Position^#^	Nearest Genes	Risk Allele	MAF	B(SE)	P-value	MAF	B(SE)	*P*-value
ALP										
rs651007	9	136153875	ABO	T	0.244	-3.045 (0.537)	1.63×10^−8^	0.254	-2.825 (0.358)	4.05×10^−15^
rs1053878	9	136131651	ABO	A	0.233	-3.029 (0.549)	3.78×10^−8^	0.240	-2.827 (0.367)	1.57×10^−14^
rs579459	9	136154168	ABO	C	0.246	-2.925 (0.537)	5.61×10^−8^	0.255	-2.885 (0.358)	9.92×10^−16^
GGT										
rs2006092	22	24995668	GGT1,SNRPD3	G	0.342	6.604 (1.000)	5.15×10^−11^	0.340	4.759 (0.594)	1.26×10^−15^
rs5751901	22	24992266	GGT1,SNRPD3	C	0.342	6.604 (0.991)	4.13×10^−10^	0.338	4.773 (0.610)	6.44×10^−15^
T.Bilirubin										
rs10929302	2	234665782	UGT1A6,UGT1A10,UGT1A9,UGT1A4,UGT1A5,UGT1A8,UGT1A3,UGT1A7	A	0.131	0.273 (0.016)	3.08×10^−64^	0.126	0.256 (0.011)	1.33×10^−116^
rs4148325	2	234673309	UGT1A6,UGT1A1,UGT1A10,UGT1A9,UGT1A8,UGT1A4,UGT1A5,UGT1A3,UGT1A7	T	0.131	0.272 (0.016)	1.01×10^−62^	0.125	0.256 (0.011)	3.07×10^−115^
rs6742078	2	234672639	UGT1A6,UGT1A1,UGT1A10,UGT1A9,UGT1A8,UGT1A4,UGT1A5,UGT1A3,UGT1A7	T	0.13	0.272 (0.016)	2.05×10^−62^	0.125	0.261 (0.011)	2.24×10^−118^
rs3755319	2	234667582	UGT1A6,UGT1A10,UGT1A9,UGT1A4,UGT1A5,UGT1A8,UGT1A3,UGT1A7	C	0.289	0.156 (0.012)	3.04×10^−36^	0.282	0.154 (0.008)	2.26×10^−75^
rs1105880	2	234601965	UGT1A6,UGT1A10,UGT1A9,UGT1A8,UGT1A7	G	0.264	0.157 (0.012)	3.21×10^−36^	0.247	0.177 (0.008)	1.18×10^−91^
rs6759892	2	234601669	UGT1A6,UGT1A10,UGT1A9,UGT1A8,UGT1A7	G	0.264	0.155 (0.012)	3.60×10^−35^	0.247	0.176 (0.009)	1.30×10^−90^
rs7586110	2	234590527	UGT1A10,UGT1A9,UGT1A8	G	0.245	0.148 (0.013)	5.13×10^−30^	0.227	0.159 (0.009)	3.84×10^−69^
rs4148323	2	234669144	UGT1A6,UGT1A1,UGT1A10,UGT1A9,UGT1A8,UGT1A4,UGT1A5,UGT1A3,UGT1A7	A	0.204	0.15 (0.014)	9.09×10^−28^	0.186	0.157 (0.010)	9.50×10^−59^
rs36075906	2	234436069	USP40	T	0.271	0.085 (0.012)	7.05×10^−12^	0.253	0.064 (0.009)	2.53×10^−13^
rs13009407	2	234652347	UGT1A6,UGT1A10,UGT1A9,UGT1A4,UGT1A5,UGT1A8,LOC100286922,UGT1A3,DNAJB3,UGT1A7	G	0.030	0.188 (0.033)	1.40×10^−8^	0.032	0.286 (0.022)	1.22×10^−39^
rs28899170	2	234604230	UGT1A6,UGT1A10,UGT1A9,UGT1A8,UGT1A7	A	0.035	0.172 (0.031)	2.99×10^−8^	0.034	0.208 (0.021)	3.25×10^−23^
rs4663580	2	234293861	DGKD	T	0.067	0.125 (0.022)	3.08×10^−8^	0.057 (0.017)	0.088	1.18×10^−7^
rs6758317	2	234168951	ATG16L1	T	0.129	0.093 (0.017)	3.24×10^−8^	0.131	0.06 (0.011)	1.01×10^−7^

#Genomic position is based on NCBI build 37

P values are adjusted for age, sex and body mass index. An additive genetic model was used.

Chr, chromosome; MAF, minor allele frequency; ALT, alanine-aminotransferase; GGT, gamma-glutamyl transferase

When examining the association with the serum GGT levels, 2 SNPs were significantly associated in the discovery set, 2 remained significant with the validation set. Among them, 2 SNPs, namely, rs5751901 and rs2006092 located in the *GGT1* gene showed strong associations with GGT (*P*-values, discovery set = 6.44 x10^-15^, and 1.26 x10^-15^, respectively; validation set = 4.13 x10^-10^, and 5.15 x10^-11^, respectively). In the regional plot in chromosome 22 is provided in Fig 3(B), which shows rs2006092 as the top SNP and nearby SNPs with the level of high LD with rs2006092.

Among the 13 SNPs that showed genome-wide significance with total bilirubin levels, rs10929302 showed the most significant association (*P*-values, discovery set = 3.08 x10^-64^, and validation set = 1.33 x10^-116^). The second most significant variants was rs6742078 (*P*-values, discovery set = 2.05 x10^-62^, and validation set = 2.24 x10^-118^), which was in high LD (r^2^ > 0.9) with *UGT1A* cluster. Highly significantly associated SNPs were located within discrete regions of the genome [Fig 3(C)]. No SNP with serum levels of ALT was associated with genome-wide significance in this study.

## Discussion

In this study, we investigated associations between liver function tests and relevant genetic loci based on GWAS. We found respectable genetic differences in this Korean population compared to previously reported European populations. First, we found that the *ABO* locus affected serum ALP levels. We also found that the *GGT1* and *SNRPD3* genes affected serum GGT levels, and *UGT1A6* and *UGT1A1* influenced the levels of BIL in this Korean population.

In this study, rs651007 and rs579459 in *ABO* gene showed significant associations with serum ALP levels. Although other various SNPs (rs657152 in European population [7], rs550057 in Japanese population [19]) within *ABO* locus on chromosome 9q34.13 were reported previously, an association between these two SNPs and ALP levels was novel. Whitfield *et al*. reported that about 15% of the genetic variance in ALP activity was associated with *ABO* blood group polymorphism in the twin study [20]. Although the exact mechanistic relationship between the *ABO* gene and ALP levels is not yet known, this relationship may depend on genetically determined variations in isoenzyme proportions among different blood types. Interestingly, the intestinal ALP in the serum is strongly involved in chylomicron formation and fatty acid metabolism might change among ABO blood group types [21]. The rs651007 and rs579459, found in this study, have been previously known to be associated with cardio-embolic stroke and large-artery atherosclerosis [22, 23]. Rs579459 is also has been found to be associated with low density lipoprotein levels and venous thromboembolism [24, 25]. Association of orthologous *ALPL* gene with ALP level was also reported in European population, which was not significant in Korean population [7].

GGT is found in the liver and biliary epithelial cells, is sensitive to hepatobiliary injury. The genetic influence for GGT estimates range 32–69% [26]. In this study, rs5751901 and rs2006092 showed significant associations with serum GGT levels. The relationship between rs5751901 and serum GGT level has been already reported in European population. It is associated with 0.21 standard deviation increase of GGT1 level [27]. In Japanese population, GGT level is associated with rs5751902, which is very close to rs5751901 in our data. This shows racial differences of regulating serum GGT levels [9]. These SNPs presumably regulate gene expression of *GGT1* [28]. *GGT1* on chromosome 22 encodes GGT, which transfers glutamyl groups linked through the gamma-carboxylic acid from peptides. It is involved in regeneration of intracellular glutathione and protection against oxidative stress [29]. Previously, several studies has reported significant associations between *HNF1A* gene on chromosome 12 and serum GGT levels [7, 28], however it was not significant in this study. Rs2006092, the location is close to rs5751901, revealed to be novel regarding to the significant association with GGT. Nonetheless, the specific role of rs2006092 and its relationship of two SNPs in *GGT1* locus need to be elucidated.

The levels of serum bilirubin are elevated in various diseases including jaundice and hemolytic disorders and the bilirubin concentration is under strong genetic regulation [6]. In American and European populations, *UGT1A1* locus (rs6742078) and *SLCO1B1* locus (rs4149056) were reported as most significant loci of serum bilirubin level [30]. In our study, rs41409056 in *SLCO1B1* locus, showed marginal significance with *p*-value = 0.0007376. In a Korean GWAS study, rs11891311 and rs4148323 variations in *UGT1A1* and the *SLCO1B3* variant (rs2417940), influenced the bilirubin levels [15]. Consistently, we found a significant association rs4148323 variations in *UGT1A1 (p*-value, 9.09E-28) and a Japanese study also reported the independence of UGT1A1*6 (rs4148323) [31]. It is most common nonsynonymous change in East Asian population and it reduces glucuronidation activity, leading to hyperbilirubinemia [32]. However, the rs2417940 in *SLCO1B3* variant did not reach genome-wide significance in this study. Top two SNPs selected in this study were rs10929302 and rs6742078. In agreement with our results, the rs10929302 which is reported to have an association with UGT1A1*93 in Korean population [33]. Another SNP, rs6742078, located in the intron1 of *UGT1A1* [34], was previously reported in a Reykjavik study cohort [30] and Korean population [15], supporting to our results. The *UGT1A* region includes nine highly similar protein-coding and four non-coding genes [35], and the SNPs that showed significant associations with serum BIL levels in this study are all located in the *UGT1A r*egion.

In European population, *CPN1* and *PNPLA3* were associated with liver enzymes [7, 8]. In Japanese population, rs2896019 in *PNPLA3* was reported [9] and the same result was published in Mexican-American ancestry GWAS [36]. However, previous GWAS study based on Korean children showed that three novel loci; *ST6GALNAC3*, *ADAMTS9*, and *CELF2* genes that were multiply associated with levels of AST and ALT [37]. These results suggest differences in regulating mechanism of liver enzymes between children and adults. However, we did not see any genome‐wide significant associations with ALT. This is most likely due to heterogeneity of the study population and sample sizes.

This is the first study that analyzed GWA of various liver function tests and SNPs in Korean population. Since the sample size is large and it was confirmed in a large number of validation set, the result has sufficient statistical power. However, this study has several limitations. First, study participants were healthy adults who voluntarily underwent health check-up at a single center. There could be regional and economical selection bias compared with entire Korean population. This could be the reason of different results compared with the study of Kang *et al* [15]. Second, the population of validation set is in same participants of discovery set. In the future, a validation study should be performed using a different population set. Finally, although liver enzymes can be influenced by alcohol intake, we could not quantitatively adjusted the amount of alcohol intake in this study.

In conclusion, *ABO*, *GGT1* and *UGT1A* gene were associated with serum concentrations of ALP, GGT and BIL, respectively in Korean population. These findings differ with respect to the identified genes and locations from prior published findings in European populations, suggesting that genetic determinants of liver enzyme levels may vary across ethnicities.

## Supporting information

S1 Table Data barcode matching. (XLSX)

## Abbreviations

ALP: alkaline phosphatase
ALT: alanine-aminotransferase
AST: aspartate aminotransferase
BIL: total bilirubin
BMI: Body mass index
GGT: gamma-glutamyl transferase
GWAS: Genome-wide association study
LFT: liver function test
SNP: single-nucleotide polymorphisms
